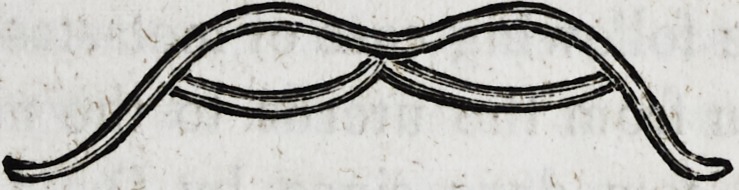# Letter from C. T. Cushman, M. D., to the Baltimore Editor

**Published:** 1849-10

**Authors:** C. T. Cushman


### ARTICLE III.

### *Letter from* C. T. Cushman, M. D., *to the Baltimore Editor.*

Columbus, Ga., *Sept.* 14*th*, 1849.

Dear Sir:—Agreeably to my promise, made you in Baltimore, in July, I will now give you a description of a method, invented by my partner, J. Fogle, of antagonizing an upper plate of teeth with the model of the opposing teeth, by means of *one cast only;* or, in other words, of getting the accurate *set* of the artificial teeth, in the absence of the patient, by a single cast, which truly shows the relative position of the plate to the lower teeth—precisely the same as when in the mouth. The following is the method of making—

### *Fogle's Single Antagonizing Cast.*

After the plate is swaged and fitted to the mouth, the clasps adjusted and fastened, and all ready for setting the teeth—build on a ridge or wall of wax, corresponding to the alveolar arch, nearly an inch in height. This is best done by warming and kneading the wax until soft, and first warming the plate over a spirit-lamp. The wax which we prefer is the composition of Dr. Griffith, of Louisville: to one pound of good yellow beeswax, add two ounces of gum mastich, and one ounce of whiting. Add the pulverized gum and whiting to the melted wax—stir until nearly cool, then mould into thin cakes, convenient for use. It may be colored red, if preferred, when melted.

Now put the plate and wax attached in the mouth, and desire the patient to press up his lower jaw gently, until the teeth meet naturally, that antagonize, and sustain the pressure and force of mastication. This done, let him gently open his mouth to its full extent, when you remove the plate with the wax, in which you will have the perfect impress of the lower teeth.

Lay the plate on the work-bench—first putting down a double fold of newspaper—with the wax uppermost. Make it lie *level*, by means of little bits of paper, if necessary, and surround it with a thick *ribbon* of *lead*, bent into a hoop, to confine the liquid plaster. This form of lead is the most convenient for this purpose, in the making of all plaster casts of the teeth, articulating models, imbedding teeth for soldering purposes; because it keeps whatever shape or position you give it, without tying or fastening. Several of these ribbons, of different thicknesses, should be kept on hand for convenient use.

Now make a thin batter of plaster—pour a little into the wax-impression, and blow upon it to get a perfect representation of the cutting edges of the lower teeth, which is an important matter; then fill up the hoop to the height of an inch, and gently shake it level with the tip of the spoon. When set, remove the hoop and paper, and while it is yet soft enough to cut easily, from the under side shave down, with a sharp knife, to the *plate*, and around the clasps, until you can remove it easily. Then take off the wax, and trim the cast a little for neatness.

You now have an impression of the plate and clasps in plaster; and just below this, and in front of it, the lower teeth appear, standing in bold relief, *in the same cast*. And it must be plainly evident to every one, that, when the plate is put back into its impress, it bears just the same relation to the plaster teeth, which it bore to the natural teeth when in the mouth. Thus—with the plate so placed, and a narrow ridge of adhesive wax on it, you may proceed to select and adapt the artificial teeth with great exactness, and more neatness and convenience than by the mouth. To admit of a plenty of wax on the plate, for this last purpose, the impress of the plate may be cut away one-half, or just so much that it will not *tilt* on its basis. If the impress of the *clasps* is left deep, this cannot well happen at all. In large, or suction plates, of course there is no apprehension on this score.

To more fully illustrate the foregoing described process, I send you, herewith, a cast of the kind, which *has been used* for the purpose intended. This will excuse its rusty appearance. You will observe a considerable piece gouged out from the impress of the plate, *opposite the second bicuspis of the lower jaw.* That was for the purpose of ascertaining the proper height and set of a *masticating block*, which, in this case, was made to antagonize with that tooth, and sustain the pressure on that side of the lower jaw—thus affording it something *firm* to rest upon, and relieving the front teeth from the danger of being knocked off—the clasp teeth from being *strained* or drawn upon—or the plate from being thrown out of position by unnatural concussion: one or all of which troubles must, otherwise, inevitably occur to an artificial piece of the kind.

As these are often required—there are so many cases where the upper front teeth, or even a whole set, are wanted, and there remain only the front teeth and two or three, or possibly all, of the bicuspides in the lower jaw; and as this *Single Antagonizing Cast* will be found the most simple and convenient guide for arranging these blocks—perhaps I ought to describe them in this connection.

Dr. Elliot, (Journal, vol. v, p. 88,) describes a similar method of overcoming the difficulties in such cases—using *small bicuspides and molares*, ground short, and square on their antagonizing surface, to meet the opposing natural teeth. Outside of these, and hiding them entirely from view, he sets a row of longer teeth—using all cuspides in the place of grinders. It will be found more convenient, however, to use *metal* blocks; and they are more agreeable to bite upon, than the surface of a mineral tooth, and, of course, not so liable to *break*. I will therefore proceed to describe to you the method of making and setting

*Fogle's Masticating Blocks.*

Supposing, then, you have a case to supply the superior teeth—say four or six—and the subject has no grinders that antagonize and afford a basis of support for the lower jaw—it then becomes indispensably necessary to set these blocks, (or something similar,) or allow the inferior incisores to touch and rest upon the plate, behind the artificials. This practice, though general, is unscientific; because it allows the jaws to approach too near each other—unnaturally shortens the physiognomy, and the dentist is obliged, if he do so, to give an unnatural *projection* to the artificial incisores. All of these difficulties are avoided by using the *masticating blocks.*

As it would be unnecessary, and a waste of material, to make them *solid*, it is best to make a *flattened tube*, and solder in a *head*. Take a piece of plate, of ordinary thickness—cut a strip as wide as the desired length of the block wanted, bend it into an oval form, whose longest diameter is a little greater than the tooth which is to strike upon it, and whose shortest diameter is sufficient that the tooth cannot well miss it, when the mouth closes naturally.

By the aid of the *Single Antagonizing Cast*, this tube may be fitted to the plate and the opposing tooth with great exactness. When so done, and stuck on with the adhesive wax, put a little plaster and sand around it, remove the wax, and solder it with a small bit. If the patient be now present, it may be tried in the mouth; and, if necessary, slight alterations may be made—as filing, compressing or *leaning* it. By filing the tube a little *beveling*, so that the posterior side shall be longest, it will be readily seen that, when the tooth strikes upon it, the tendency will be to drive the plate more firmly into its position. Now bevel the inside of the tube, and bevel a head to let into and fit it, and solder this and the block *permanently;* or the soldering of these last may be deferred, and done by the same heat which fastens the teeth to the plate. After the block has got its final set and adjustment, the dentist may proceed to select and arrange the teeth as usual.

In such a case as I have instanced, it may be neces- to set one or more blocks on *each side of the mouth*, to sustain the pressure equally. When this is advisable, a firm tooth should be selected for each—one that is likely to last well. A plate so made can be worn with great ease and comfort, as the pressure from the inferior jaw is *equally* divided over the palatal surface covered by the plate—and the artificial teeth may be set in *natural position*, and so as to completely hide the blocks from view.

In consideration of the simplicity of the *Single Antagonizing Cast,* and its various and *valuable* uses, I am now forced to question your opinion of the ordinary *double* articulation cast—that *they* are "the most accurate and convenient antagonizing model *that can possibly be* obtained."—(Principles and Prac., 3d ed., p. 663.)

I next proceed to describe to you

*Fogle's Temporary Fastenings for Clasps.*

These may be regarded as a very important improvement upon any hitherto known method of getting the *set* of clasps to plates of artificial teeth, or plates for correcting irregularities, defective palates, &c.

The usual method is, to get the set by means of wax holding the clasp to the plate, or be guided by the plaster cast alone. Some dentists practice one way, some the other. The first method is tedious, and often impracticable. If the fastening-teeth stand a little *leaning,* the wax becomes disturbed in withdrawal from the mouth, and the relative position of the clasp is changed. This, too, may happen in more favorable cases—so frail is the tenure by which they are connected, and the operator not perceive it until too late for remedy—after it is soldered.

In the other method, it is found impossible to get that *precision* of set by the plaster model, which is attainable by this method, for various reasons: one is, if the fastening-teeth stand a little *leaning* in the mouth, (as they almost invariably do after the approximal tooth is removed,) the model does not give a correct fac-simile of the mouth, because the wax impression had a "drag" from these teeth in withdrawal, which, of course, was transmitted to the plaster cast. Secondly, the model is a hard and unyielding substance, while the natural parts, to which the plate is to be adapted, are quite the reverse—soft and elastic.

By this method, one may as easily get the *accurate* and *exact* set of the clasps, as *by the mouth.* First, make your clasps by the plaster model; leave the *ends* straight for the present. Here I would observe, that in fastening to the second, and sometimes *third* molares, the clasp has to be *curiously* shaped, to set closely around the tooth, and *hold on.* A *straight* clasp bent to the circumference of the tooth, if a *dens sapientiæ,* would only touch at *two or three points,* owing to the peculiar shape—the sides being generally short, rounded or *bulging,* and *beveled,* particularly on the posterior side—and it could not be made to *stay on.*

An excellent way to overcome this difficulty is, to fit a clasp of *thin lead* to the model-tooth, by bending it and cutting away the edges until it has an accurate fit all round. Then straighten this, and lay it upon the material from which the clasp is to be cut, (we use eighteen carat gold, generally a little thicker than the plate;) mark around it with a sharp point, and *cut by the pattern.* This gives a very perfect fit.

After the clasps are made, cut a strip of plate about a line in width, and about five-eighths of an inch in length. Place the clasp on a coal, and one end of the strip flatwise, in juxtaposition with the upper part of the lingual surface of it: apply borax and a bit of solder to the point of union, and make fast in a moment. Now place both on the model, and bend the other end of the strip until it touches the plate, forming a *bow* or semi-circle. File it to make a *point,* and shorten it, if necessary—apply borax and solder as before, and make fast in another moment.

You will perceive that the clasps are now fitted and fastened *by the model;* but, on trying the plate in the mouth, they may be *swung around, raised up, lowered, twisted,* or set in any desirable position, to give them an *exact* and easy adaptation to the teeth. The *set* that is given them will be maintained so firmly by these *temporary fastenings,* that it will not easily get changed, in the subsequent handling of the piece. The *permanent* soldering may now be given them—placing the piece on the plaster model for the purpose. It is well to shave away the plaster under the joint, to facilitate the flow of solder through to the under side. Or this may be deferred, and done by the same heat which unites the teeth to the plate. We generally prefer the former way. In getting the *accurate* set of the clasps, if they have been pushed away from the plate, so as to leave a *space* between; this may be filled with chips or clippings of plate and solder, and all united. After this is done, the *bows* are cut off, the solder filed and scraped down and polished off.

In very *difficult* cases of adjustment—as where the clasp-teeth stand *leaning*—where you have to fasten to the second or third molares, it will be found still more advantageous to pursue *this* plan, viz. after soldering one end of the strip to the clasp, and having bent the other to touch the plate when on the model; put both in their proper place *in the mouth;* then, with a sharp-pointed instrument, indicate the point where the bow touches the plate. Place them on the model again, *adjust the end of the bow to the point marked,* confine it there, and solder fast. This will save considerable *twisting* and subsequent adjustment in the mouth.

Every practical dentist knows the vexatious difficulty usually attendant upon getting the *clasps* perfectly fitted to the teeth and plate, in such cases as I have just specified. Indeed, I have seen many such, where, I venture to assert, that a perfect, or even a *tolerable* fit, could not possibly be obtained by either of the methods *generally* pursued. This method, however, will effectually surmount all such, and the *temporary fastenings* can be put on in fifteen minutes, by an expert workman.

So valuable do I consider this method, I never venture now, in the simplest cases, to set the clasps permanently *by the model;* for, when so done, it is almost invariably found, on trial in the mouth, that they do not touch the teeth on the lingual surface—a space intervenes, which forms a receptacle for agents that must tend to act upon and destroy the tooth.

To better illustrate the foregoing description, I accompany it with a plaster-model of a mouth, and a raised plate, and clasps attached, by the *temporary fastenings*—completed only to that stage when it is *ready for trial in the mouth,* and permanent adjustment. The plate is of *copper,* gilded by the electro-galvanic process. (Comstock, Philosophy, p. 348.) This is a duplicate of one of our recent cases, and, as we regard it, one of the *difficult* ones I have specified—the fastening-teeth being the second molares, and in a leaning position, occasioned by the loss of the adjacent teeth.

You will observe, in this plate, an artificial *ruga* raised—it is for the purpose of *stiffening* it. It also answers the purpose of an *air-chamber,* in a degree—which of course helps to keep the plate steady and firm in its position. This ridge is made on the plaster-model, while it is yet damp from withdrawal from the wax—by tracing on a *very thin* solution of plaster, with a small camel's-hair pencil, and afterwards trimming with a penknife. The idea was first suggested by Dr. Ide, of Columbus, Ohio, (Dent. Reg., vol. i, p. 209.) He uses *whiting*, but that is more liable to get crumbled off in casting. The whole natural rugæ of the mouth may be so enlarged artificially—or rather their fac-similes—in large plates, with the advantages accruing which he specifies: increased stiffness in the plate—relief of the natural rugæ from pressure—increased adhesiveness of the plate.

I know one dentist who invariably shaves his plaster-models *perfectly smooth*, with a sharp knife, on their palatal surface. He thus removes every vestige of the natural rugæ, and makes his dies and stamps his plates, (very thick ones,) accordingly. This may be called the *reverse* system of practice—making *nature* subservient to *art*—fitting the mouth to the plate, instead of the plate to the mouth. His *philosophy* is, that unless this is done, these membranous folds "will *change their position* afterwards, and throw the plate out of set." (!) Although he is an "*M. D.*" dentist, "he ought," as an accomplished dentist remarked, to whom I related this *unique* practice, "to study his Anatomy."

The conseqeunce of wearing such ill-fitting and unyielding plates, with broad, heavy clasps, *fitted by the model*—as I have witnessed in his patients, is, to press these folds unnaturally flat; they have to sustain a pressure which almost entirely deprives them of circulation. The gums are literally *torn* from their natural adhesion to the necks of the teeth, by this unequal pressure. Foreign deposits of food, mucus and tartar are admitted between the teeth and gums—acute, and finally, chronic inflammation of the periosteal tissue is induced and established.*

* Since writing the foregoing, I have read Dr. Austen's article "On the Use of Clasps," in the Journal, (July number,) in which he urges the well-known objections to their use, and inquires of the profession whether a substitute may not be generally adopted, in the employment of suction-plates.

I do not believe that the use of *clasps* for sustaining plates of artificial teeth, can ever be wholly dispensed with. The only substitute known at this time, or that seems likely *to be* known, is the one he proposes; and to that there are as many objections to its practical use, as to properly constructed clasps. Those which Dr. A. specifies must ever remain.

I recently saw a well made upper set, belonging to a distinguished gentleman—the *fourth* that he had got made, by as many distinguished dentists at the north, wherever his extensive inquiry led him to find them. It was sustained by the *air-chamber,* and held firmly; but the prominence so *impeded his articulation,* and made him so uncomfortable at all times, that he was impelled to cut it out himself. He is now obliged to have the *fifth* piece made, and called on us for the purpose.

Again—a person who wants but a single tooth set—say a superior lateral incisor, would not willingly be burdened with a large plate, covering the whole palatine arch, and having this *in-the-way-prominence* impeding articulation to a greater or less degree. An atmospheric-pressure plate, with air-chambers made by the *raised method* which I have specified, would, by reason of its great surface, be but little less objectionable.

One of the greatest triumphs of art, in a case where it is required to supply a defect or loss of nature, would be a *concealment* of its resource. It is next to impossible for a person wearing a full-size plate, to conceal its broad and brilliant surface from the occasional view of his associates—and those who are obliged to have recourse to art, to *preserve their good looks,* are not generally ambitious of having the fact published, either through others or *themselves.* This fact, calling upon the inventive faculties of man for remedy, has suggested the *enameling process,* to hide the gold from sight; and it has been practiced, to some extent, in times past and present. Indeed, a *perfect enameling process* seems to me *now* to be a great *desideratum,* for the covering of *small plates* as well as large. But as attempts at this have hitherto failed of accomplishing the end desired, they have not been made available.

If, for a single tooth, or two or three teeth, one were obliged to wear a large air-chamber plate, with its insuperable objections, which have been stated, the remedy would be almost as bad as the loss; and, I apprehend, most persons would be deterred from adopting it, after fairly understanding the case.

By employing the artificial raised-work, a *thin* plate may be made as stiff and unyielding as one of nearly double its thickness without it. The object in this case, of which you have the duplicate, was principally to keep the plate from springing—it being very long. A ridge was therefore raised on the plaster model, much in the shape of a *Cupid's bow*, extending from the middle of the palate vault, back to the fastening-teeth. The *ornamental* is thus combined with the *useful.*

This simple method will be found to fully answer the purpose of that recommended by Dr. Roper, of Philadelphia, to be employed when long plates are required—which is, to extend a narrow piece of plate *across* from one side of the mouth to the other, just under, and adapted to, the palatine arch.—(Harris, "Principles and Practice," 3d ed., p. 687.) That is much more troublesome to accomplish—to get the plate and the cross-piece to set closely at the same time. I have done so, however, and in a very satisfactory manner; but best by first taking a large plate, as I would for an atmos-

*Clasps,* then, seem to me to be indispensable; and this admitted, our energies should be directed to *perfecting* their construction and mode of application. The *temporary fastenings* are a great stride towards this end, for most of the objections which Dr. A. urges against clasps, vanish when they are *perfectly adjusted* to the teeth and the plate.

A plate whose clasps are *set by the model,* when inserted in the mouth, and subjected to the pressure of mastication, *settles* in its yielding bed; then the clasps must necessarily exert a considerable tractive force upon the teeth to which they are attached, and produce evil results. But by this new method, the plate is put in the mouth and *pressed up firmly,* and the clasps *then* adjusted to the position which they will be made to assume in *practical use.* This, then, in my opinion, obviates most of the objections hitherto maintained against their use.

My opinion is further supported by the practical *results* of this method, in a practice of some years—the plates so adjusted being yet worn with perfect ease to the mouth and teeth to which they are attached.

pheric-pressure piece, and, after swaging it, cutting out a crescent-shaped piece from the center of the palatine arch. This gave the same result as fitting and soldering on a separate cross-piece, and with more exactness and less trouble.

To further illustrate the *raised method* of stiffening small plates, I send you also a small plate, with the two superior central incisores mounted—accompanied by the plaster-model of the mouth. This is another duplicate of one of our recent cases, and the plates were made according to the plan I have described.

Although this accompanying plate is very thin, light, and of *copper*, which is soft, you will perceive, on taking the two ends between your thumb and index finger, and testing it by pressing them together, that it is very stiff and unyielding. This is entirely owing to the artificial ridge, or *Cupid's bow,* which is raised upon it. Furthermore, you will observe in this case, two additional ridges raised on the plate, in the shape of *crescents,* which face the concave side of the largest curves in the *bow,* and the ends of which touch it—so that both together describe an elliptical circle at that point—near the centre of the palatine vault—one on each side.

These crescents *doubly* stiffen it, and prevent still more the extremities of the *bow* from approaching each other, when pressure is applied to them. They were made, as they can be in most cases, by following mostly the natural rugæ of the mouth; and, indeed, the most of the middle part of the *bow* may also be made by following the *natural prominences.* In this case, the elliptical circles of the *bow* and *crescents* comprise the greater portion of the natural rugæ within their circumference. Simple as this method is, in my opinion nothing could be devised which would more effectually, and in so neat and *ornamental* a manner, add to the stiffness of a light plate. Still, the subject is open to investigation and improvement; and I hope each member of the profession will contribute his mite to the general treasury.

Nothing particular is claimed for the *workmanship* of the piece on which the teeth are mounted and clasps set—our only object being to illustrate the *method* described.

I remain, Sir,
Yours, with high respect,
C. T. CUSHMAN.

## Figures and Tables

**Figure f1:**